# Translation without eIF2 Promoted by Poliovirus 2A Protease

**DOI:** 10.1371/journal.pone.0025699

**Published:** 2011-10-07

**Authors:** Natalia Redondo, Miguel Angel Sanz, Ewelina Welnowska, Luis Carrasco

**Affiliations:** Centro de Biología Molecular “Severo Ochoa” (CSIC-UAM), Universidad Autónoma de Madrid, Madrid, Spain; University of Minnesota, United States of America

## Abstract

Poliovirus RNA utilizes eIF2 for the initiation of translation in cell free systems. Remarkably, we now describe that poliovirus translation takes place at late times of infection when eIF2 is inactivated by phosphorylation. By contrast, translation directed by poliovirus RNA is blocked when eIF2 is inactivated at earlier times. Thus, poliovirus RNA translation exhibits a dual mechanism for the initiation of protein synthesis as regards to the requirement for eIF2. Analysis of individual poliovirus non-structural proteins indicates that the presence of 2A^pro^ alone is sufficient to provide eIF2 independence for IRES-driven translation. This effect is not observed with a 2A^pro^ variant unable to cleave eIF4G. The level of 2A^pro^ synthesized in culture cells is crucial for obtaining eIF2 independence. Expression of the N-or C-terminus fragments of eIF4G did not stimulate IRES-driven translation, nor provide eIF2 independence, consistent with the idea that the presence of 2A^pro^ at high concentrations is necessary. The finding that 2A^pro^ provides eIF2-independent translation opens a new and unsuspected area of research in the field of picornavirus protein synthesis.

## Introduction

Viral proteases play an important part both in the generation of mature viral proteins and in the modulation of cellular functions [1], [2]. Three proteases have been described in different picornavirus species: 2A^pro^, L^pro^ and 3C^pro^ [3].This last protease, 3C^pro^, and its precursor 3CD^pro^, are present in all picornavirus species and are responsible for most proteolytic cleavages of the viral polyprotein . The three proteases are capable of cis-autoproteolysis, by which they are excised from the viral polyprotein. It seems reasonable to think that the main purpose of PV 2A^pro^ and FMDV L^pro^ is to modify cellular functions. Indeed, both proteases bisect eIF4G at a position close to each other. The cleavage site of PV 2A^pro^ on eIF4GI is located between amino acids 681–682 [4]. Bisection of eIF4G takes place soon after PV infection, leading to inhibition of cellular translation, while the bulk of PV proteins is synthesized at late times when virtually all eIF4G has been proteolyzed. Thus, hydrolysis of eIF4G by PV 2A^pro^ inhibits the canonical mechanism of translation, which is cap-dependent and promotes a non-canonical mechanism in which eIF4E and cap recognition are not necessary [4]. Apart from this cleavage, PV 2A^pro^ can hydrolyze other cellular proteins, although the exact degradome for this protease has still not been defined. Some of these hydrolytic events associated with PV 2A^pro^ involve the proteolysis of nucleoporins, thereby altering RNA and protein trafficking between nucleus and cytoplasm [4]. Therefore, PV 2A^pro^ blocks cap-dependent translation upon eIF4G cleavage and interferes with mRNA export to the cytoplasm; both events abolish cellular gene expression and abrogate cellular responses to viral infection.

The translation initiation factor eIF4G is a large polypeptide which can interact with several cellular and viral proteins. Two forms of eIF4G encoded by two different genes are known, eIF4GI and eIF4GII [5]. The exact functioning of each of these two forms in the process of translation remains unclear, although it has been suggested that these forms are functionally interchangeable. Three regions have been distinguished in eIF4G, each of which harbours the interaction sites with several cellular proteins. Binding of eIF4E and eIF4A to eIF4G gives rise to the formation of the eIF4F complex [6], [7]. Interaction of eIF4F with mRNA may take place directly or indirectly. Thus, eIF4E directly binds to the cap structure present at the 5′ end of mRNAs, while eIF4A unwinds the secondary structure of the mRNA leader sequence. In addition, eIF4G itself interacts with picornavirus IRESs by means of its central domain [8], [9], [10]. Apart from these direct interactions of the eIF4F complex with mRNAs, eIF4G also interacts with eIF3 and PABP, both of which also can directly bind to mRNA. Joining of the eIF4F complex to the 40S ribosomal subunit is mediated by the interaction between eIF4G and eIF3. Therefore, during the initiation of translation, eIF4G plays a pivotal role as a scaffolding molecule organizing the architecture of different initiation factors, mRNA and the preinitiation complex [6], [7]. The central role of eIF4G in mRNA translation makes it a key target for a variety of animal viruses. Indeed, modulation of eIF4G activity by viral proteins may be essential for cytopathic viruses to control translation. Calicivirus as well as some picornavirus and retrovirus species encode proteases that hydrolyze eIF4G during infection [4], [11], [12], [13]. Alternatively, a number of viral proteins are able to interact with eIF4G, modulating its activity. This is the case of rotavirus NSP3 [14], influenza virus NS1 and PB2 [15], [16] and adenovirus 100 K protein [17]. Cleavage of eIF4G by picornavirus proteases 2A^pro^ or L^pro^ leads to the stimulation of IRES-driven translation [4]. Pestova and colaborators demonstrated that the central domain of eIF4G together with eIF4A interacts with EMCV IRES and promotes the formation of the preinitiation complex [18], [19]. Consistent with this finding, the C-terminal fragment or even the core domain of eIF4G suffices to promote IRES-driven translation both in vivo and in cell free systems [20], [21].

eIF4F activity is regulated in eukaryotic cells by extra- and intracellular signals through phosphorylation [4]. eIF4E activity is also controlled by phosphorylation by the protein kinase Mnk1 or by interaction with eIF4G, which is modulated by eIF4E binding proteins (4E-BPs) [7]. Phosphorylation also represents the most important mechanism to regulate eIF2 activity. Factor eIF2 is composed of three subunits, known as α, β and γ [6], [22]. Several kinases target eIF2α leading to phosphorylation of Ser-51 residue. The function of eIF2 is to bind Met-tRNA_i_ and GTP to form the ternary complex Met-tRNA_i_-eIF2-GTP, which interacts with the 40S ribosomal subunit, establishing the interaction between the initiator AUG codon with the anticodon present in Met-tRNA_i_ [6], [7]. The hydrolysis of eIF2-bound GTP is promoted by eIF5, while the eIF5B–GTP complex facilitates recruitment of the 60S subunit to the 48S initiation complex. This joining promotes that the translation initiation factors except for eIF5B–GTP and eIF1A are displaced. The eIF2-GDP complex is recycled to eIF2-GTP by the activity of the recycling factor eIF2B. Phosphorylation of eIF2α impairs the GDP-GTP recycling catalyzed by eIF2B. Therefore, the ternary complex Met-tRNA_i_ -eIF2-GTP is not generated and thus, binding of this complex to the 40S ribosome is hampered. Even partial phosphorylation of eIF2 can lead to substantial abrogation of translation. Some reports suggested that this factor remained unphosphorylated after poliovirus (PV) infection [23], [24], while other workers found substantial eIF2 phosphorylation under the same conditions after PV infection, particularly at late times [25], [26]. Of interest, Protein Kinase R (PKR) becomes highly activated, yet it is hydrolyzed in PV-infected cells although this hydrolysis is not directly executed by any of the PV proteases (2A or 3C) [25], [26], [27]. All these findings pointed to the idea that active eIF2 was necessary to sustain picornavirus translation. In contrast to this idea, we described recently that several picornaviruses do not require active eIF2 at late times of infection [28], similar findings have been reported for PV-infected cells [29]. In the present work we provide evidence that cleavage of eIF4G by PV 2A^pro^ in mammalian cells modifies the requirement for eIF2 in translation directed by picornavirus IRESs. Thus, cleavage of eIF4G by PV 2A^pro^ establishes a mechanism for IRES-driven translation that is cap- and eIF2 independent. These unexpected findings indicate that PV 2A ^pro^ induces eIF2 independence IRES-driven translation by a mechanism that is still unknown.

## Results

### Dual mode for translation of PV RNA

**Figure 1:**
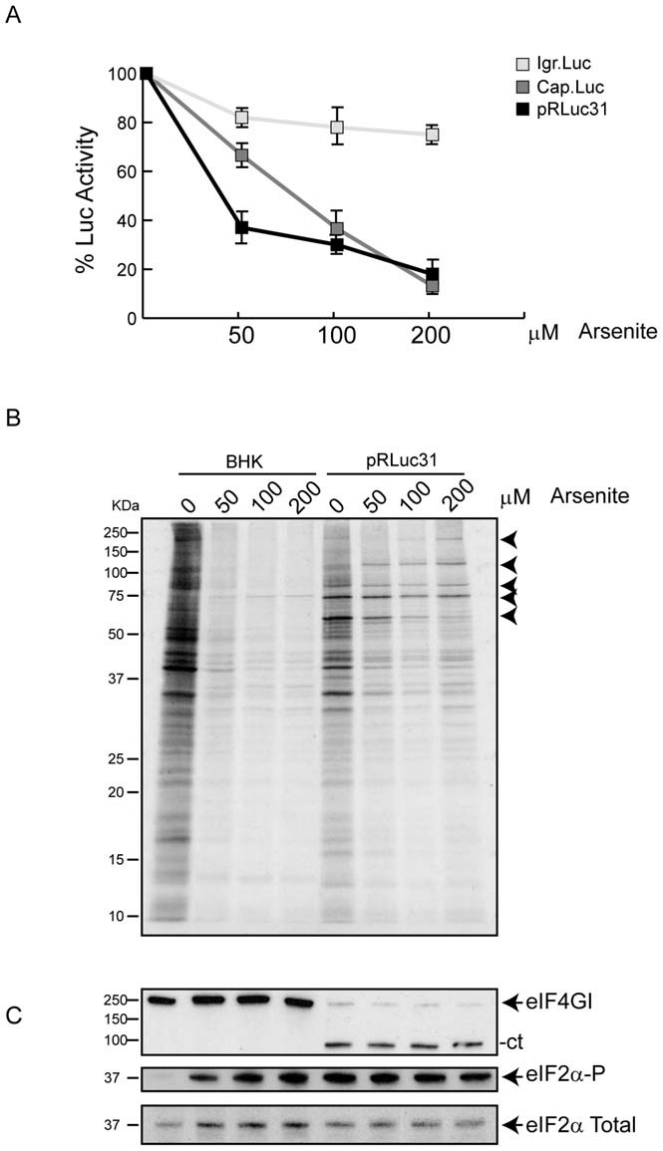
Effect of eIF2 phosphorylation induced by Ars on PV protein synthesis. A) Cap-luc, PV replicon-luc or CrPV IGR-luc mRNAs synthesized *in vitro* by T7 RNA polymerase were electroporated in BHK-21 cells and seeded in DMEM (10% FCS). Different amounts of Ars (0, 50, 100 and 200 µM) were added and cells were incubated for 60 min before harvesting to analyze luc. Error bars indicate standard deviations (SD) obtained from three measurements of each sample. B) BHK-21 cells were electroporated with RNA of PV replicon. At 7 hpe cells were treated with different concentrations of Ars and labelled with [^35^S]Met/Cys for 45 minutes. Samples were analyzed by SDS-PAGE (17.5%) followed by fluorography and autoradiography. Arrows indicate viral proteins. C) eIF4GI, eIF2α and phosphorylated eIF2α were detected by western blot.

Some viral mRNAs, when they are translated in virus-infected cells, have different requirements for eIFs as compared to cell-free systems or transfected cells [30], [31]. This is the case of Sindbis virus 26S mRNA, which does not require intact eIF4G [32] or active eIF2 [33] for translation in the infected cells, whereas these eIFs are necessary to initiate protein synthesis on this viral mRNA in cell-free systems [31]. Although it is generally accepted that picornavirus RNA needs eIF2 to initiate translation, there is some evidence that this factor can be phosphorylated at late times of infection [26], [34]. Indeed, recently we found that several picornaviruses exhibit this dual mode for translation of the viral mRNA [28]. So we hypothesized that this factor might be dispensable at late times in the PV life cycle, when the bulk of viral proteins are being synthesized. To test this possibility, eIF2 was inactivated by treating culture cells with Ars to induce phosphorylation of eIF2α. This compound induces oxidative stressand has been widely used to inactivate eIF2 [35], [36], [37]. A PV replicon (pRLuc31) containing the luciferase (luc) gene replacing the viral structural proteins was used [38]. As controls, cells were also electroporated with Cap-luc or CrPV IGR-luc mRNAs [28], [29] and at 1 hpe cells were treated with different concentrations of Ars (0, 50, 100 and 200 µM) for 1 h. Electroporation of these RNAs into BHK-21 cells gives rise to luc synthesis from the beginning of transfection. This early luc synthesis was produced by translation of the input RNA and was drastically blocked by Ars treatment in the case of PV replicon to an extent similar to that found with a capped mRNA whereas CrPV IGR-luc was inhibited by only 20% (Figure 1A). At 7 hours post transfection (hpt), PV proteins can be detected by radioactive labelling because cellular protein synthesis is abrogated. Notably, Ars treatment has little inhibitory effect on the translation of PV RNA, whereas translation of cellular mRNAs was blocked by about 90% under the same conditions (Figure 1B). It should be noted that Ars interferes with the cleavage of the PV polyprotein as already observed [28], [29]. Certainly, Ars treatment led to eIF2α phosphorylation, both in control and in PV RNA transfected cells. Of interest was that phosphorylation of eIF2α was also found in PV-replicating cells in the absence of Ars (Figure 1C, middle panel). In addition, cleavage of eIF4G was progressively observed along the PV replication cycle (Figure S1B, upper panel). Analysis of eIF2α phosphorylation throughout the time course of PV replication provides evidence that this factor became phosphorylated at times when PV protein synthesis was maximal and eIF4G had been cleaved (Figure S1A). These findings demonstrate that PV RNA exhibits a dual mechanism for the initiation of translation as regards the participation of eIF2. At early times, before viral RNA replication has occurred, active eIF2 is required to translate PV RNA, whereas this factor is dispensable at late times when massive production of viral proteins is taking place.

### Analysis of PV non-structural proteins that confer eIF2 independence for viral RNA translation

**Figure 2:**
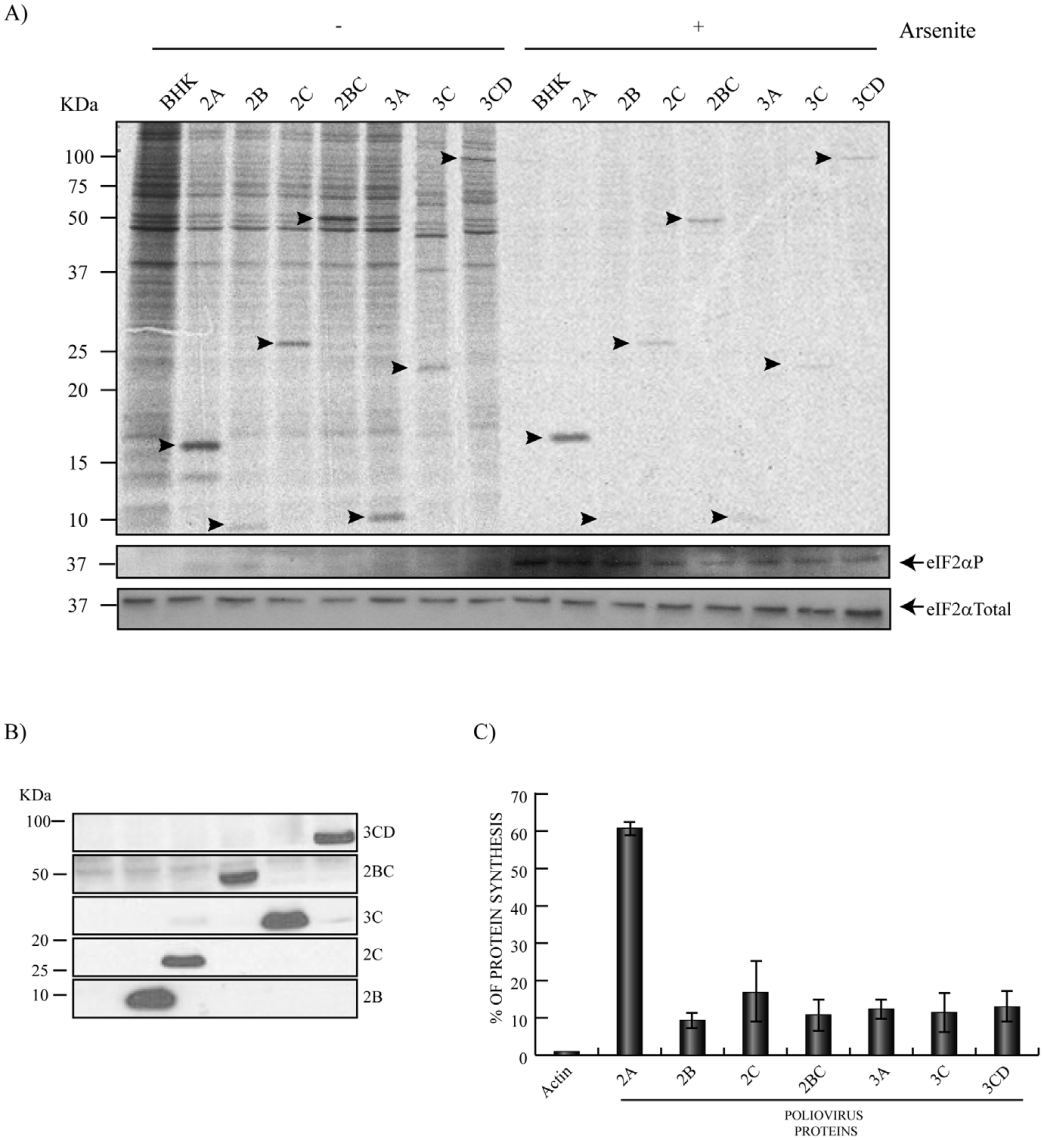
Individual expression of PV non structural proteins. Action of eIF2 phosphorylation. BHKT7 cells were transfected with pTM1 plasmids encoding different PV non-structural proteins and were(+) or were not(−) treated with Ars. A) After 2 hpt cells were pre-treated with 200 µM Ars for 15 minutes and then labelled with [^35^S]Met/Cys for 45 minutes in presence of the inhibitor. Then, samples were processed by SDS-PAGE (17.5%), fluorography and autoradiography. Western blot of total eIF2α and phosphorylated eIF2α using the same samples is shown at the bottom of this panel. B) PV non-structural proteins were detected by western-blot. C) The percentage of actin (*) and each PV protein synthesis was estimated by densitometric scanning of the corresponding band (arrows) from three independent experiments. Error bars indicate SD.

Since the PV replicon tested above only encodes PV non-structural proteins in addition to luc, we reasoned that perhaps extensive individual expression of each PV non-structural protein might establish conditions similar to those observed during PV replication. Under these conditions of high PV protein synthesis, active eIF2 might not be necessary to translate PV RNA. Moreover, it may be that synthesis of a single PV protein was able to confer eIF2-independence for IRES-driven translation. To test this possibility, the system used was the BHKT7 cell line, which stably expresses T7 RNA polymerase. Although this polymerase is devoid of capping activity, transfection of plasmids encoding different PV non-structural proteins under the control of a T7 promoter gives rise to extensive translation of mRNAs bearing a picornavirus IRES sequence. The different pTM1 constructs encoding for each PV non-structural protein were transfected into BHKT7 cells and the synthesis of PV proteins was analyzed by radioactive labelling in presence or absence of Ars (Figure 2A), as well as by western blot (Figure 2B). As shown in Figure 2A, all PV proteins can be clearly detected by radioactive labelling in absence of Ars. Strikingly, PV 2A^pro^ is extensively synthesized even in the presence of Ars, when eIF2α has become phosphorylated. Thus, Ars inhibited cellular translation more than 90%, whereas the synthesis of PV 2A^pro^ was blocked by only 35% (Figure 2C). The inhibition of the other PV non-structural proteins by Ars treatment was around 80% (Figure 2C) and in some cases such as 2B, 3A and 3C their synthesis was almost undetectable (Figure 2A). Therefore, the expression of one individual PV protein, 2A^pro^, can confer independence from active eIF2 for picornavirus–IRES-driven translation.

### Translation of mRNAs containing different picornavirus IRESs in the presence of 2A^pro^: Requirement for active eIF2α

**Figure 3:**
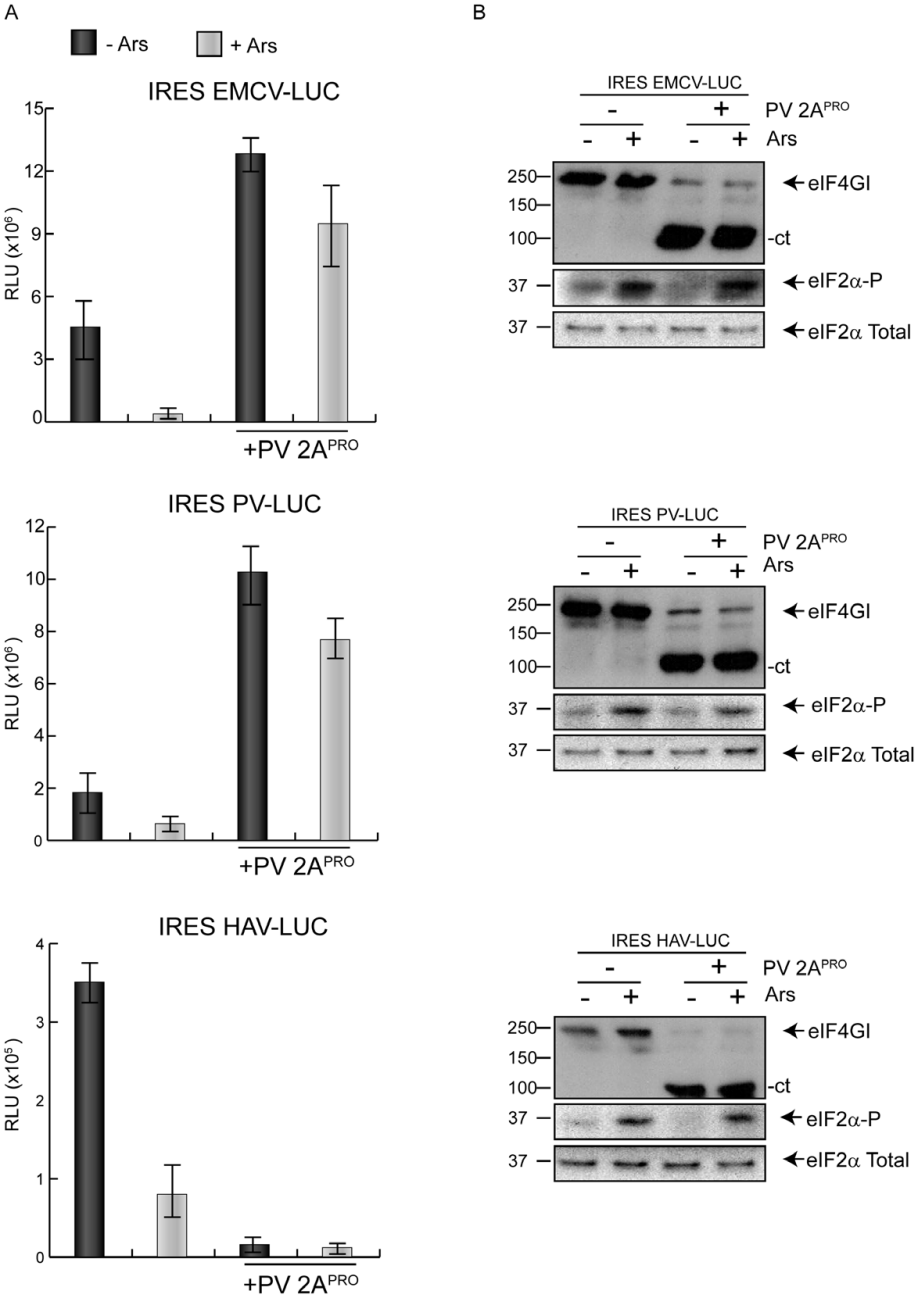
Inhibition of translation directed by PV or EMCV IRES by Ars. Rescue by PV 2A^pro^. A) BHKT7 cells were transfected with plasmids containing EMCV IRES-luc, PV IRES-luc or HAV IRES-luc alone or co-transfected with pTM1-2A. At 2 hpt cells were treated or not with Ars for 1 hour. Then, cells were harvested and lysated in luciferase buffer and luc activity was measured (as described in Materials and Methods) and represented from at least three independent experiments. Error bars indicate standard deviation (SD). B) eIF4GI, eIF2α and phosphorylated eIF2α were detected by western blot.

Our next goal was to assess whether PV 2A^pro^ was able to confer eIF2 independence *in trans* for the translation of other mRNAs bearing a picornavirus IRES. To this end, the synthesis of luc directed by EMCV-, PV- and HAV-IRES was tested in the presence or absence of Ars, when culture cells did or did not co-express PV 2A^pro^. The synthesis of this protease in culture cells rescues the inhibition of Ars by about 70% when EMCV or PV IRESs are tested (Figure 3A). Notably, translation driven by HAV IRES is abolished when co-expressed with PV 2A^pro^ in presence or absence of Ars. These results agree well with previous studies indicating that HAV IRES requires the intact form of eIF4F for functionality [39], [40], [41]. Similar results were obtained in the human hepatoma Huh7-T7 cell line (Figure S2). Therefore, translation of luc mRNA bearing different picornavirus IRESs is hampered when eIF2α phosphorylation is induced by Ars. Of interest, PV 2A^pro^ is able to confer translatability to EMCV and PV IRESs, but not to HAV IRES under these conditions.

**Figure 4:**
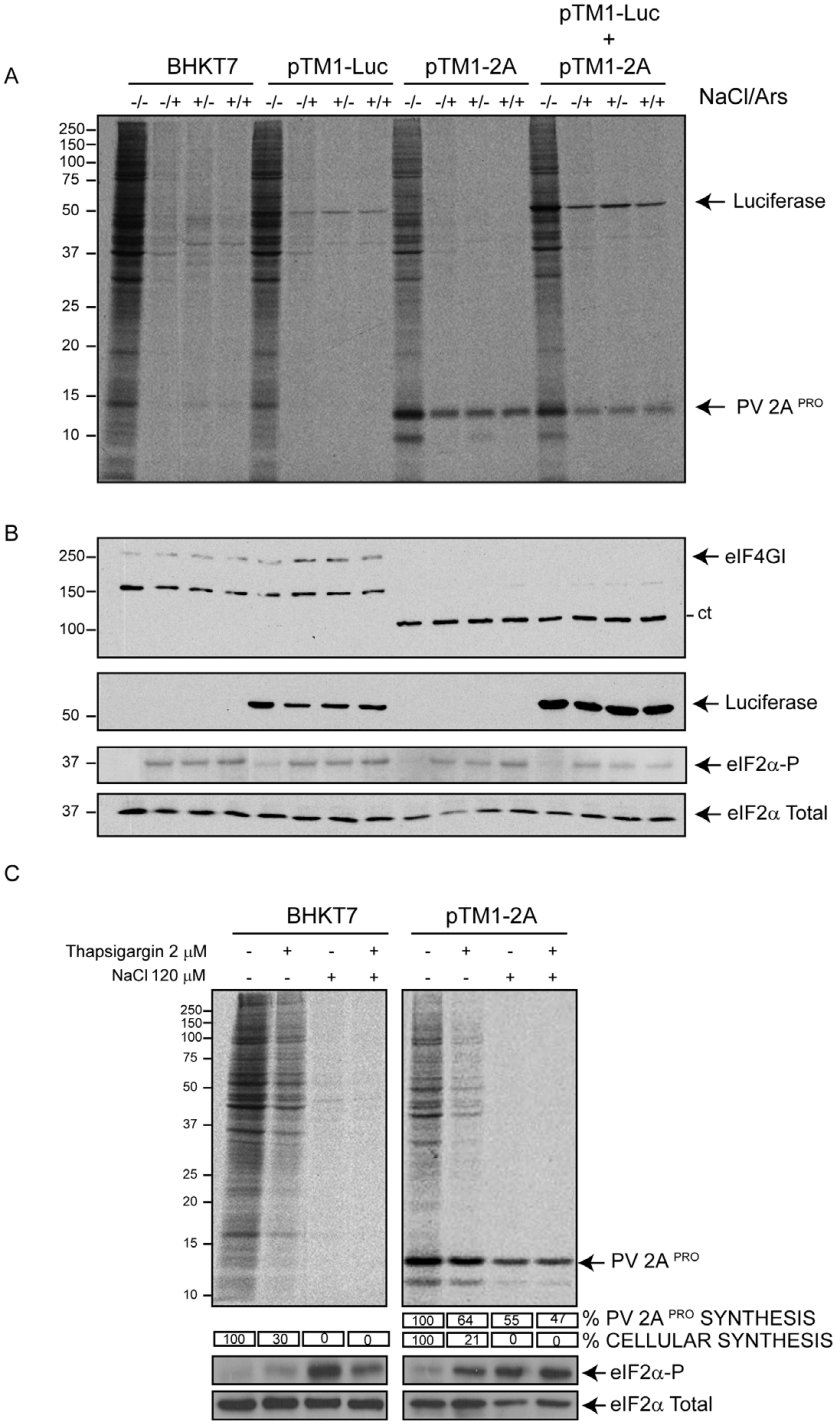
Effect of different inducers of eIF2α phosphorylation on IRES-driven translation. A) BHKT7 cells were transfected with pTM1-luc, pTM1-2A or both. At 2 hpt cells were pretreated with Ars(−/+), NaCl(+/−) or both(+/+) for 15 minutes and then labelled with [^35^S]Met/Cys for 45 minutes in presence of the inhibitors. After labelling, the proteins were analyzed by SDS-PAGE(17.5%), fluorography and autoradiography. B) Western blot analysis of samples from panel A using anti-eIF4GI, anti-Luc, anti-phosphorylated eIF2α and anti-total eIF2 antibodies. C) Cells were mock transfected or transfected with pTM1-2A. At 2 hpt cells were pretreated with 1 µM Tg or additional 120 mM NaCl or both for 15 minutes and then were labelled with [^35^S]Met/Cys for 45 minutes in presence of the inhibitors. After labelling, proteins were analyzed by SDS-PAGE, fluorography and autoradiography. Numbers below each lane indicate the percentage of cell protein (*) and PV 2A^pro^ synthesis in cells treated with inhibitor compared with untreated cells quantified by densitometry of the corresponding bands. A western blot using antibodies against eIF2α and phosphorylated eIF2α was performed.

In addition to Ars, there are other treatments for inducing phosphorylation of eIF2α, such as incubation of culture cells with hypertonic medium or Thapsigargin (Tg) [30], [42]. To assay the effect of these treatments on IRES-directed translation, BHKT7 cells were transfected with pTM1-luc, pTM1-2A or co-transfected with pTM1-luc and pTM1-2A. Extensive inhibition of cellular translation was observed when cells were treated either with Ars, hypertonic medium or both (Figure 4A). Inhibition of luc synthesis also occurs when pTM1-luc is transfected alone. However, when PV 2A^pro^ is synthesized under these conditions, significant levels of IRES-2A translation are detected (Figure 4A). Hypertonic medium promotes eIF2α phosphorylation, particularly when combined with Ars (Figure 4B). A similar conclusion can be drawn when cells are transfected with pTM1-2A and treated with Tg (Figure 4C) or with dithiothreitol (results not shown). These findings support the idea that translation of IRES-2A mRNA is resistant to different compounds and treatments that induce phosphorylation of eIF2α when high levels of PV 2A^pro^ are synthesized.

PV infection induces partial PKR degradation, as well as its phosphorylation which correlates with increased eIF2α phosphorylation as infection progresses. To test whether PV 2A^pro^ expression diminished the amount of PKR in our culture cells, a western blot analysis was carried out using specific antibodies against PKR. The levels of this enzyme were similar in cells that either did or did not express PV 2A^pro^ (Figure S3).

### Proteolytic activity of PV 2A^pro^ is necessary to confer eIF2 independence

**Figure 5:**
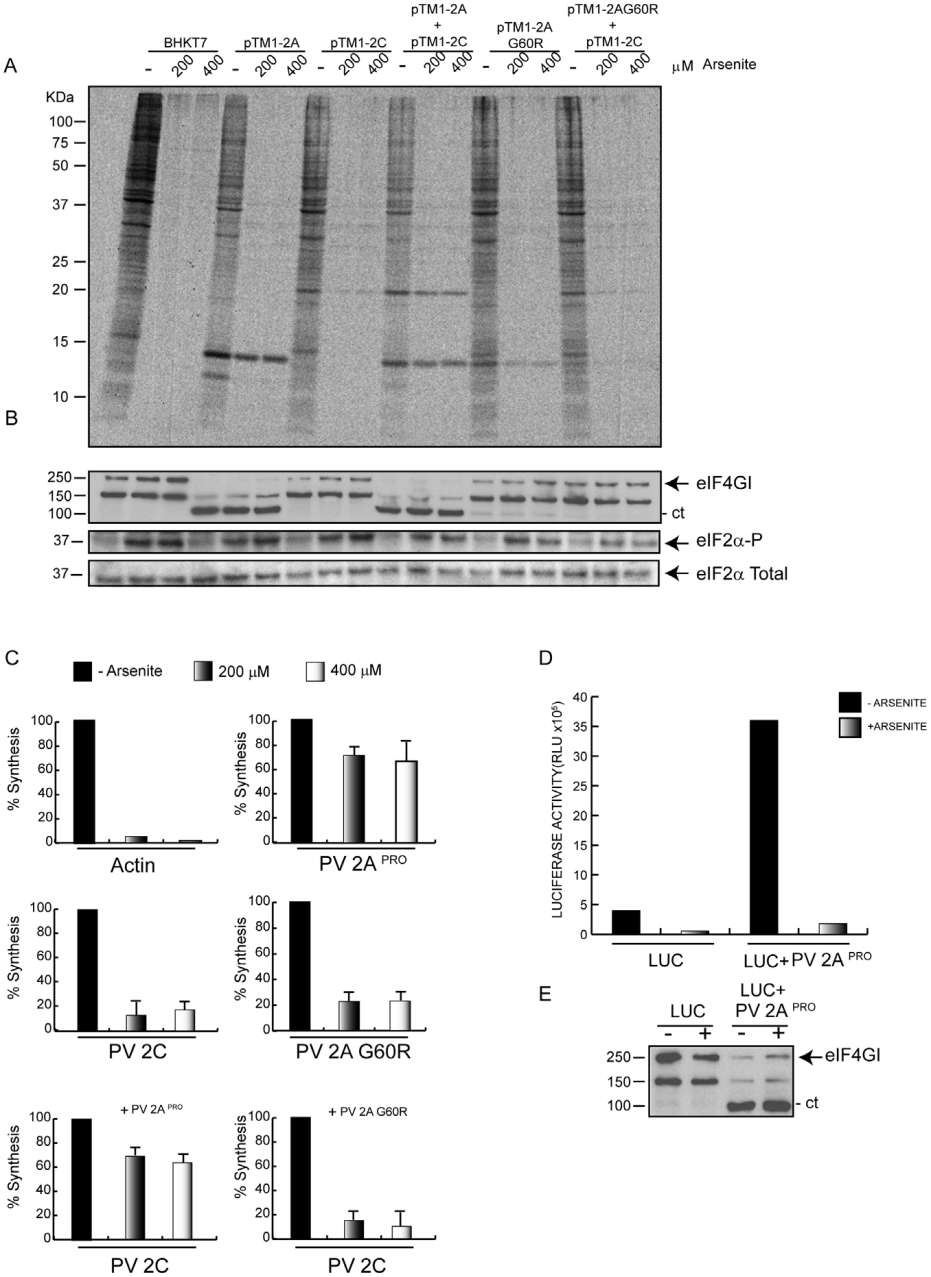
Proteolytic activity is necessary for eIF2α independent translation. BHKT7 cells were transfected or co-transfected with either pTM1-2A or pTM1-2A G60R, which encodes for an inactive 2A^pro^, and pTM1-2C. A) At 2 hpt cells were treated with different Ars concentrations and incubated with [^35^S]Met/Cys for 45 minutes. Samples were analyzed by SDS-PAGE (17.5%), fluorography and autoradiography. B) eIF4GI, phosphorylated eIF2α and total eIF2α of the same samples were detected by western blot. C) The percentage of cellular and viral protein synthesis, measured by densitometric scanning of the corresponding band from at least three independent experiments, is shown. Upper panels show the synthesis of actin (representing cellular protein synthesis), 2A wt, 2C and 2A G60R when they are expressed by separate. Lower panels show the synthesis of PV 2C protein alone, either in presence of 2A wt or in presence of 2A G60R. All data are shown as the mean ±SD of at least three independent experiments. D) BHKT7 cells were first transfected with IRES-2A mRNA. After 2 hpt, cell monolayers were washed and incubated in fresh medium (DMEM plus 5% FCS) for 1 h to accomplish the cleavage of eIF4G. Then, pTM1-luc was transfected during 2 h, afterwards transfection medium was removed and cells were incubated in fresh medium and after 15 minutes were treated or not with 200 µM Ars during 1 h . Finally, cell monolayers were harvested in luciferase buffer and luc activity was measured and represented. E) Cleavage of eIF4GI of the samples used in panel D was detected by western blot.

Next, we wished to examine the effect of eIF2 phosphorylation on IRES-driven translation when eIF4G remained uncleaved. To this end, a PV 2A^pro^ variant bearing a point mutation (G60R) devoid of eIF4G cleavage activity [43], [44] was employed. In this case, plasmid pTM1-2C was co-transfected with pTM1-2A or pTM1-2A (G60R). As a control, the same constructs were expressed alone. PV 2A^pro^ and 2C synthesis were analyzed both in the presence or absence of Ars. Cellular translation was abolished by Ars, as well as the synthesis of PV 2C and PV 2A (G60R) (Figure 5A). By contrast, PV proteins 2C and 2A^pro^ are still synthesized in presence of Ars, when PV 2A^pro^ is expressed alone or when PV 2C is co-expressed with PV 2A^pro^. The labelled proteins separated by SDS-PAGE were quantified by densitometric analyses (Figure 5C). Synthesis of PV 2C was inhibited by only 30–35% in presence of Ars and PV 2A^pro^, while this inhibition was of 85–90% when 2A^pro^ (G60R) was present (Figure 5C, lower graphs). This result indicates that the presence of high levels of 2A^pro^ in the absence of eIF4G cleavage does not induce eIF2 independence for IRES-directed translation.

Another approach to abolishing eIF4G cleavage is to use PV 2A^pro^ inhibitors. Addition of methoxysucciniyl-Ala-Ala-Pro-Val-chloromethylketone (MPCMK) strongly blocks cleavage of eIF4G [45] even when high levels of PV 2A^pro^ are synthesized in BHKT7 cells. The presence of this 2A^pro^ inhibitor abolishes eIF2 independence for translation of picornavirus IRES (see below). In conclusion, cleavage of eIF4G (together with other putative cellular protein (s)) accomplished by active 2A^pro^ is necessary for this phenomenon.

### Cleavage of eIF4G is not sufficient to provide eIF2-independent translation

The only known direct effect of PV 2A^pro^ on translation is that this protease cleaves eIF4G, leading to stimulation of picornavirus RNA translation [8]. Thus, it is possible that eIF2-independent translation is the consequence of the generation of the two eIF4G fragments after bisection by PV 2A^pro^. Alternatively, it is possible that in addition to eIF4G, other host proteins could be hydrolyzed by this protease providing eIF2-independent translation. Moreover, the presence of PV 2A^pro^ itself could be necessary, and in this scenario 2A might play an IRES trans-acting role. To distinguish between these possibilities different experiments were conducted. Initially, we tested the effect of Ars on EMCV IRES-driven translation in the presence of low or high levels of PV 2A^pro^. Low amounts of this protease are produced in cells when in vitro synthesized IRES-2A mRNA is transfected [46], whereas high levels of 2A^pro^ are found in culture cells using the system described in this work. Under both conditions, eIF4G becomes extensively cleaved. Addition of Ars to cell cultures transfected with IRES 2A mRNA and later with plasmid encoding IRES-luc (pTM1-luc) profoundly blocked translation, irrespective of whether or not PV 2A^pro^ was present (Figure 5D). Under those conditions, eIF4G was almost totally cleaved and both eIF4G fragments were present (Figure 5E), but the levels of 2A^pro^ are low and do not confer eIF2-independence. By contrast, when high amounts of PV 2A^pro^ are synthesized in BHKT7 cells, Ars has little inhibitory effect on EMCV IRES-driven translation. These findings support the notion that the presence of eIF4G fragments (or the cleavage of other cellular proteins) is necessary but not sufficient to confer eIF2 independence for picornavirus IRES-driven translation.

**Figure 6:**
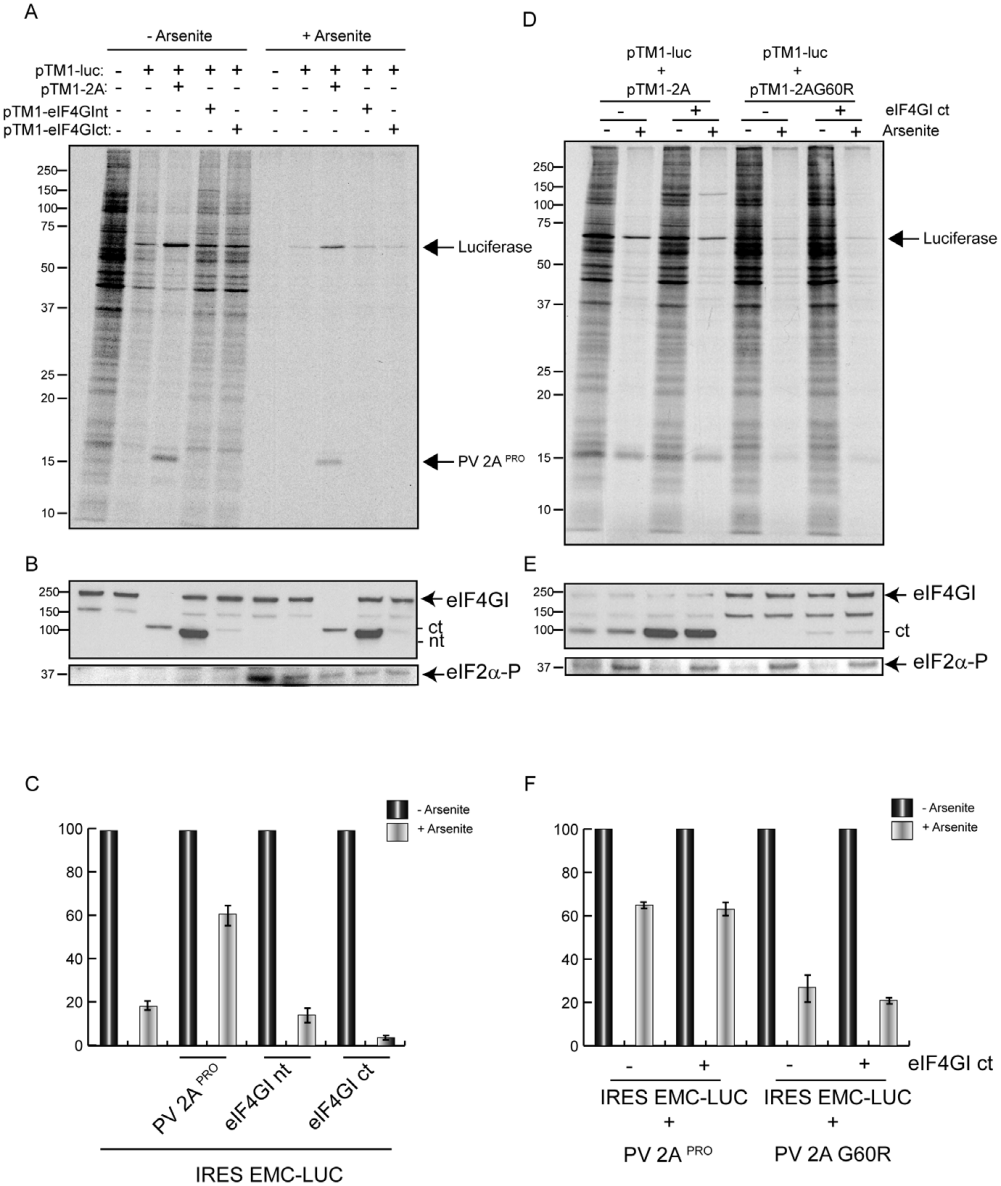
Influence on IRES-directed translation of expression of eIF4G fragments. pTM1-luc was co-transfected with a combination of the next plasmids: pTM1-2A , pTM1-eIF4Gnt and pTM1-eIF4Gct. A) At 2 hpe cells were pre-treated with 200 µM Ars for 15 minutes and then labelled with [^35^S]Met/Cys for 45 minutes in presence of the inhibitor. Samples were processed by SDS-PAGE (17.5%) followed by fluorography and autoradiography. B) The amount of eIF4GI, eIF2α and phosphorylated eIF2α of the samples were detected by western blot. C) The percentage of luc synthesis, measured by densitometric scanning of the corresponding band, was represented. Error bars indicate SD from at least two independent experiments. D) BHKT7 cells were co-transfected with pTM1-luc and either pTM1-2A wt or pTM1-2A G60R. To each mixture, plasmid expressing c-terminal fragment of eIF4GI was or was not added. At 3 hpt samples were first pretreated with Ars for 15 minutes and then radiolabeled with [^35^S]Met/Cys for 45 minutes and were or were not treated with Ars. Samples were then processed by SDS-PAGE (17.5%) followed by fluorography and autoradiography. E) eIF4GI, eIF2α and phosphorylated eIF2α were detected by western blot. F) The percentage of luc synthesis, measured by densitometric scanning of the corresponding band, was represented. Error bars indicate SD from at least three independent experiments.

To provide further support for this conclusion, the two eIF4G fragments generated by PV 2A^pro^ cleavage were synthesized in BHKT7 cells by transfection of the corresponding pTM1 plasmids. These two fragments correspond to the cleavage products of eIF4G accomplished by PV 2A^pro^. The synthesis of each fragment was detected by immunoblotting (Figure 6B). Synthesis of luc from EMCV -luc was sensitive to Ars even when cells expressed either of the eIF4G fragments (Figure 6A). A densitometric analysis of the corresponding products synthesized is represented in Figure 6C. The inhibition of luc synthesis by Ars is around 40% when PV 2A^pro^ is present but is greater than 80% when luc is expressed either alone or with the N-terminal or C-terminal fragments of eIF4GI. In conclusion, the idea that the C-terminus fragment of eIF4GI interacts with EMCV IRES thereby allowing mRNA to be translated without eIF2 is not supported by these results. In fact, we demonstrate that high levels of PV 2A^pro^ must be present to translate picornavirus RNA when eIF2α is phosphorylated.

In addition, we tested whether the presence of high levels of both the inactive mutant 2A G60R and the carboxy fragment of eIF4G can switch translation to an eIF2-independent mode. When PV 2A^pro^ is or is not synthesized together with the C-fragment of eIF4GI, Ars has little effect on translation driven by EMCV IRES (Figure 6D). In fact, the synthesis of the C-terminal fragment of eIF4G is stimulated when co-expressed with PV 2A^pro^. The percentage of luc synthesis is about 70% in presence of Ars when is co-expressed with PV 2A^pro^ with or without the eIF4GI C terminal fragment (Figure 6F). However, luc synthesis is notably diminished by Ars to around 20% when luc is synthesized either with PV 2A (G60R) alone or with PV 2A (G60R) together with the C-terminal fragment of eIF4GI. These observations indicate that to achieve resistance to eIF2 phosphorylation, both the cleavage of eIF4G (or other cellular protein (s)) and the synthesis of high levels of active PV 2A^pro^ are necessary.

**Figure 7:**
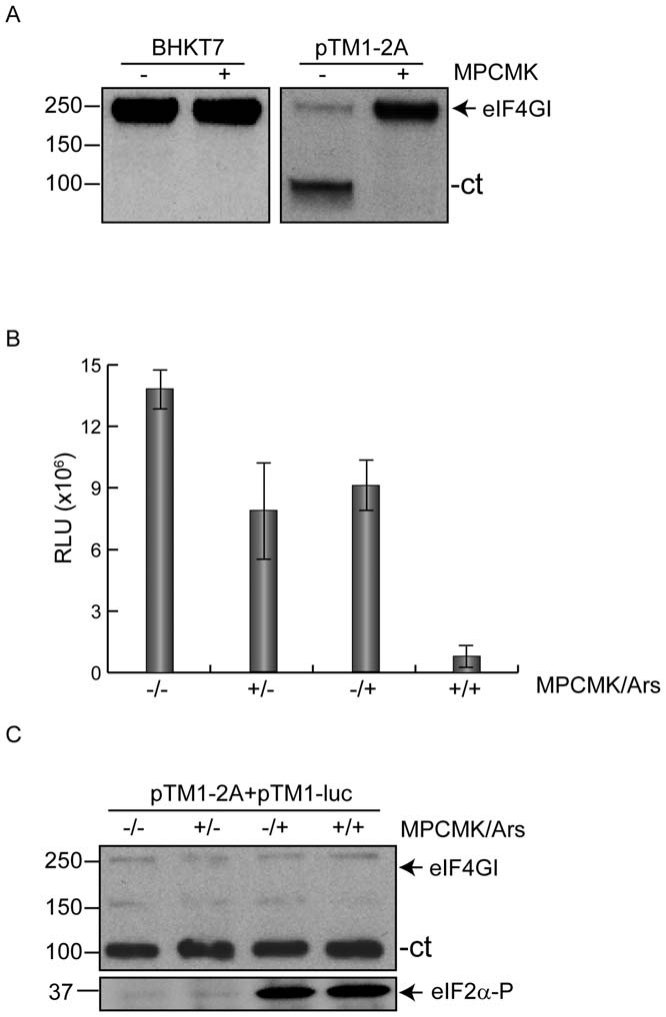
Active PV 2A^pro^ is necessary for eIF2 independence. A) BHKT7 cells were transfected or not with pTM1-2A and at the same time, in the mixture of transfection, cells were incubated without or with (−/+) 750 µM MPCMK. The transfection mixture was removed and cell monolayers were incubated for 1 h with or without (−/+) the inhibitor. To analyze the inhibitory effect on the proteolytic activity of PV 2A^pro^, eIF4GI was detected by western blot. B) BHKT7 cells were transfected with pTM1-2A. After, cell cultures were incubated for 1 h at 37°C, then cells were transfected with pTM1-luc for 30 minutes. Afterwards, transfection mixture was removed and cells were incubated with or without 750 µM MPCMK (+/−), with 200 µM Ars (−/+) or both of them (+/+) for 1 h. Finally, cells were harvested and lysated in luciferase buffer and luc activity was measured and represented from at least three independent experiments. Error bars indicate SD. C) The samples obtained in panel B were used to examine eIF4GI by western blot. Phosphorylated eIF2α also was detected by western blot.

Two possibilities can be envisaged to account for the above findings. One is that PV 2A^pro^ cleaves a putative cellular protein other than eIF4G when present at high levels. This putative cleavage would be necessary to confer eIF2 independence. Another possibility is that active 2A^pro^ must be present to observe this phenomenon. To distinguish between these two possibilities, cells were transfected with pTM1-2A and after 1 h of incubation, when eIF4G and other putative cellular proteins had been cleaved, pTM1-luc was transfected in the presence or absence of MPCMK, which is an inhibitor of the proteolytic activity of 2A^pro^ (Figure 7A) . Addition of this inhibitor, even after PV 2A^pro^ has exerted its proteolytic activity renders IRES-driven translation dependent on active eIF2 (Figure 7B). These findings therefore demonstrate that cleavage of other putative cellular protein is not involved in this phenomenon. In conclusion, both cleavage of eIF4G and active PV 2A^pro^ are required to render IRES driven translation independent of eIF2.

## Discussion

Progressive inactivation of eIF2 by phosphorylation takes place upon infection of culture cells with some PV variants and other picornaviruses [24], [26], [34]. This eIF2 inactivation was previously thought to play a role in the abrogation of cellular and viral protein synthesis at late times of infection, since the prevailing idea was that picornavirus RNA translation needs active eIF2. Our present data demonstrate that significant phosphorylation of eIF2α is found in PV-replicating cells from about 3 hpi. Moreover, induction of substantial eIF2 phosphorylation by Ars has little effect on PV protein synthesis, while cellular translation is drastically abolished under these conditions. Our present results are in good agreement with recent findings indicating that several picornaviruses, including PV, can translate their mRNA when eIF2α is phosphorylated at late times of infection [28], [29]. The claim that cleavage of eIF5B by PV 3C^pro^ as responsible for eIF2-independent translation [29] is not supported by our results illustrating that upon the individual expression of each PV protein only 2A^pro^ is endowed with this activity. If this is so, the mechanism of picornavirus RNA translation may be more similar to the situation reported for flaviviruses, since translation of their viral RNAs may not use eIF2, when this factor is absent [36], [47], [48]. We also have demonstrated that the individual expression of PV 2A^pro^, but not other PV non-structural proteins, is sufficient to render picornavirus IRES-driven translation independent for active eIF2. This effect is observed both in *cis* and in *trans* on mRNAs bearing picornavirus IRES elements. These mRNAs are little affected upon phosphorylation of eIF2 induced by different inhibitors when high levels of PV 2A^pro^ are synthesized.

PV 2A^pro^ is a multifunctional protease that targets a number of cellular processes, including translation [4], [49]. Indeed, this viral protease bisects eIF4G thereby disrupting cap-dependent translation of the vast majority of cellular mRNAs. By contrast, this modification of eIF4G enhances PV protein synthesis [8]. Most evidence indicated that simple cleavage of eIF4G is not sufficient for this stimulation [21], [50]. Indirect evidence points to a direct activity of 2A^pro^ in PV RNA translation [49], [51], thus the actual presence of 2A^pro^ together with cellular protein cleavage would be necessary to stimulate IRES-driven translation. The C-terminal fragment of eIF4G is able to replace the entire factor in cell free systems [18]. However, overexpression of this fragment in intact cells does not stimulate picornavirus IRES-driven translation [21], [50], [52]. Consistent with these findings, our present observations indicate that the expression of either the N-or C-terminus fragments of eIF4G in our system does not stimulate translation directed by EMCV IRES. Our findings support the concept that for eIF2 independence during initiation of IRES-containing mRNAs, both cellular protein cleavage and the presence of high levels of PV 2A^pro^ are necessary.

It is most striking that after several decades of studies on the mechanism of picornavirus translation, the possibility that eIF2 may not participate in this process has not been uncovered. It is generally thought that translation on picornavirus RNA requires active eIF2 [22]. This mechanism has been supported by many studies using cell free systems. However, to our knowledge the idea that eIF2 might not participate in the initiation of translation of PV RNA in the infected cells has not been investigated. Notably, PV translation is blocked by Ars during the early period of infection, supporting the notion that PV RNA exhibits a dual mode for its translation, as occurs for instance with Sindbis virus 26S mRNA [31]. Therefore, PV RNA may follow two different mechanisms for the initiation of translation: one canonical mechanism using entire eIF4G and eIF2 early during infection and another mechanism at the late phase of the virus life cycle. This last mechanism does not require intact eIF4G or active eIF2. Remarkably, the presence of PV 2A^pro^ alone suffices to provide independence from active eIF2.

The new and unsuspected findings that the translation of mRNAs bearing picornavirus IRESs takes place when eIF2 has been inactivated by phosphorylation open a future area for research in the field of picornavirus translation. In addition, the fact that PV 2A^pro^ can switch picornavirus RNA translation from an eIF2 dependent mechanism to a different mode of initiation establishes the first molecular basis for this phenomenon. Future work will target the elucidation of potential cellular proteins or factors that can replace eIF2 during picornavirus RNA translation. It is even possible that in the infected cells or in the presence of PV 2A^pro^ the IRES structure is sufficient to signal the initiation codon in a way akin to that described for Cricket paralysis virus IGR IRES [53], [54]. Several reports have appeared about the potential replacement of eIF2 by other cellular proteins for the translation of hepatitis C virus (HCV) RNA [48], [55], but these experiments have always been carried out in *in vitro* systems in the absence of any viral protein. Some authors believe that eIF5B can replace eIF2 for the translation of HCV RNA in reconstituted cell free systems [36]. A recent report suggests that ligatin (also known as eIF2D) could replace eIF2 for HCV, but not EMCV RNA translation [48]. Although cell free systems have been very useful for unravelling the mechanisms of protein synthesis, they may provide some artefacts. Therefore, the observations found in *in vitro* systems must be contrasted with the situation present in intact cells and in virus-infected cells.

## Materials and Methods

### Cell Cultures

Baby Hamster Kidney (BHK-21 and clon BSR-T7/5, designated as BHKT7) cells [56] and Huh7-T7 (Human Hepatoma,) were used in this work. Cells were grown at 37°C in Dulbecco's Modified Eagle's Medium (DMEM) supplemented with 5% or 10% fetal calf serum (FCS) and non-essential amino acids. Cells BHKT7 were additionally provided with Geneticin G418 (Sigma) on every third passage at a final concentration of 2 mg ml^−1^ cell culture medium. For Huh7-T7 cells the medium was supplemented with Zeocin (5 µM).

### Plasmids and transfections

The pTM1-derived plasmids containing the poliovirus proteins were described in detail earlier [44], [57], [58], [59] . The constructs pKs.Luc and pTM1-luc have been already described [60]. The pTM1-eIF4GInt and pTM1-eIF4GIct were constructed using the pcDNA3 HAeIF4G-I [5] as DNA template. In the case of N-terminal fragment, were used the primers 5′NcoI4GInt: GCGCGCCCCATGGCCACGCCTTCTCAG and 3′BclI4GInt: GCGCTGATCATTAGCCAAGGTTGGCCAAG and, in the case of C-terminal the primers used were 5′EcoRI4GIct: GCGCGCAAATTCGGACAACCCTTAGC and 3′BclI4GIct: CCGCTGATCAGTTGTGGTCAGACTCCTCC. The PCR products were digested with NcoI/BclI or EcoRI/BclI respectively and inserted into the pTM1, previousy digested with the same enzymes. BHKT7 cells were transfected using Lipofectamine 2000 (Invitrogen). Cells were transfected or co-transfected with 1 µg of plasmid DNA or a mixture comprising 1 µg of each plasmid; in the case of RNA transfection, 2 µg of 2A mRNA were added plus 2 µl of Lipofectamine per well in Opti-mem medium (Invitrogen) for 2 hours at 37°C. After 2 hours, Lipofectamine was removed, and the cells were supplemented with fresh medium containing 5% FCS. BHK-21 cells were electroporated with *in vitro* synthesized mRNAs using as DNA templates the PV replicon, pKS.Luc or T7 Rluc ΔEMCV IGR-Fluc (this plasmid was employed to obtain CrPV IGR-luc mRNA). To obtain Cap-luc mRNA from pKS.luc, an m^7^G(5′)ppp(5′)G cap analog was added to the transcription mixture. Transcription reactions were carried out with T7 RNA polymerase (Promega) according to the manufacturer's instructions. For transfection, subconfluent BHK cells were harvested, washed with ice-cold phosphate-buffered saline (PBS), and resuspended at a density of approximately 2.5×10^6^ cells/ml in the same buffer. Subsequently, 40 µg of *in vitro* transcribed RNA were added to 0.8 ml cell suspension and the mixture was transferred to a 4-mm cuvette (Bio-Rad). Electroporation was performed at room temperature by generating one pulse at 350 V and 975 µF using a Gene Pulser II apparatus (Bio-Rad). Finally, cells were diluted in DMEM supplemented with 10% FCS and seeded onto culture plates.

### Inhibitor treatments and analysis of protein synthesis by radioactive labelling

BHKT7 cells were transfected or co-transfected with the corresponding plasmids. At different time points, after two hours of incubation with transfection mixture, cells were pre-treated with 200 µM sodium arsenite (Ars) (Riedel-de Haën) or 2 µM Thapsigargin (Tg) (Sigma) for 15 min at 37°C, or left untreated. Next, proteins were radiolabelled for 45 min with [^35^S]Met/Cys (Promix; Amersham Pharmacia) in methionine/cysteine-free DMEM in the presence or absence of the corresponding concentration of Ars or Tg. Finally, cells were collected in sample buffer, boiled for 4 min and analysed by SDS-PAGE (17,5%) and fluorography. Protein synthesis was quantified by densitometry using a GS-710 calibrated Imaging Densitometer (Bio-Rad). In the case of NaCl treatment, a methionine/cysteine-free DMEM with a final concentration of 265 (120+145) mM NaCl was used. Proteins were then radiolabelled for 45 minutes. Finally, cell monolayers were resuspended in sample buffer and processed as described above.

### Western blotting

Transfected cells were collected in sample buffer, boiled and processed by SDS-PAGE. After electrophoresis, proteins were transferred to a nitrocellulose membrane as described previously [61]. To detect PV non-structural proteins, specific rabbit polyclonal antibodies [43], [61], [62] were used at dilution 1∶1000. To detect eIF4GI a rabbit antibodies mix against the N-terminal and C-terminal portion of this protein [63] were used at dilutions of 1∶1000. Polyclonal rabbit antibodies against eIF2α (Santa Cruz biotechnologies) and phosphorylated eIF2α (Cell Signaling) were used at a 1∶1000 dilution. Rabbit antisera were raised against firefly luciferase (Promega). Incubation with primary antibodies was performed for 2 h at room temperature, and then the membrane was washed three times with PBS containing 0.2% Tween-20 and incubated for 1 h with horseradish peroxidase-conjugated anti-mouse (Promega) or anti-rabbit IgG antibodies (Amersham) at a 1∶5000 dilution. After washing three times, protein bands were visualized with the ECL detection system (Amersham).

### Measurement of Luciferase Activity

Cells were recovered in a buffer containing 25 mM glycylglycine (pH 7.8), 0.5% Triton X-100 and 1 mM dithiothreitol. Luc activity was determined using *luciferase assay system* (Promega) and Mononlight 2010 apparatus (Analytical Luminescence Laboratory) as described previously [11], [12].

## Supporting Information

Figure S1 **Kinetics of PV Replicon.** BHKT7 cells were transfected withPV replicon. A) Protein synthesis was determined by labelling with [^35^S]Met-Cys for 45 minutes every two hours from 1 to 7 hpt. B) Western blot analysis of the samples obtained in panel A using anti-eIF4G, anti-Luciferase and anti-phospho-eIF2α.
(TIF)

Figure S2 **Rescue of picornavirus IRES translation by PV 2A^pro^ in Huh7-T7 cells.** A) Hepatoma cells were transfected with plasmids encoding EMCV IRES-luc, PV IRES-luc or HAV IRES-luc alone or co-transfected with pTM1-2A. At 2 hpt cells were treated or not with 200 µM Ars for 1 hour. Then, cells were harvested and lysated in luciferase buffer and luc activity was measured and represented as percentage from at least three independent experiments. Error bars indicate SD. B) eIF4GI were detected by western blot.
(TIF)

Figure S3 **Effect of 2A^pro^ on PKR.** BHKT7 cells were mock- or transfected with pTM1-2A in presence or absence of Ars. Protein kinase RNA-activated (PKR) was detected by western blot.
(TIF)
