# Supplementary figures and images for: Translation without eIF2 Promoted by Poliovirus 2A Protease
Source: PLoS One. 2011 Oct 7;6(10):e25699. doi: 10.1371/journal.pone.0025699 (PMC3189197; doi:10.1371/journal.pone.0025699)

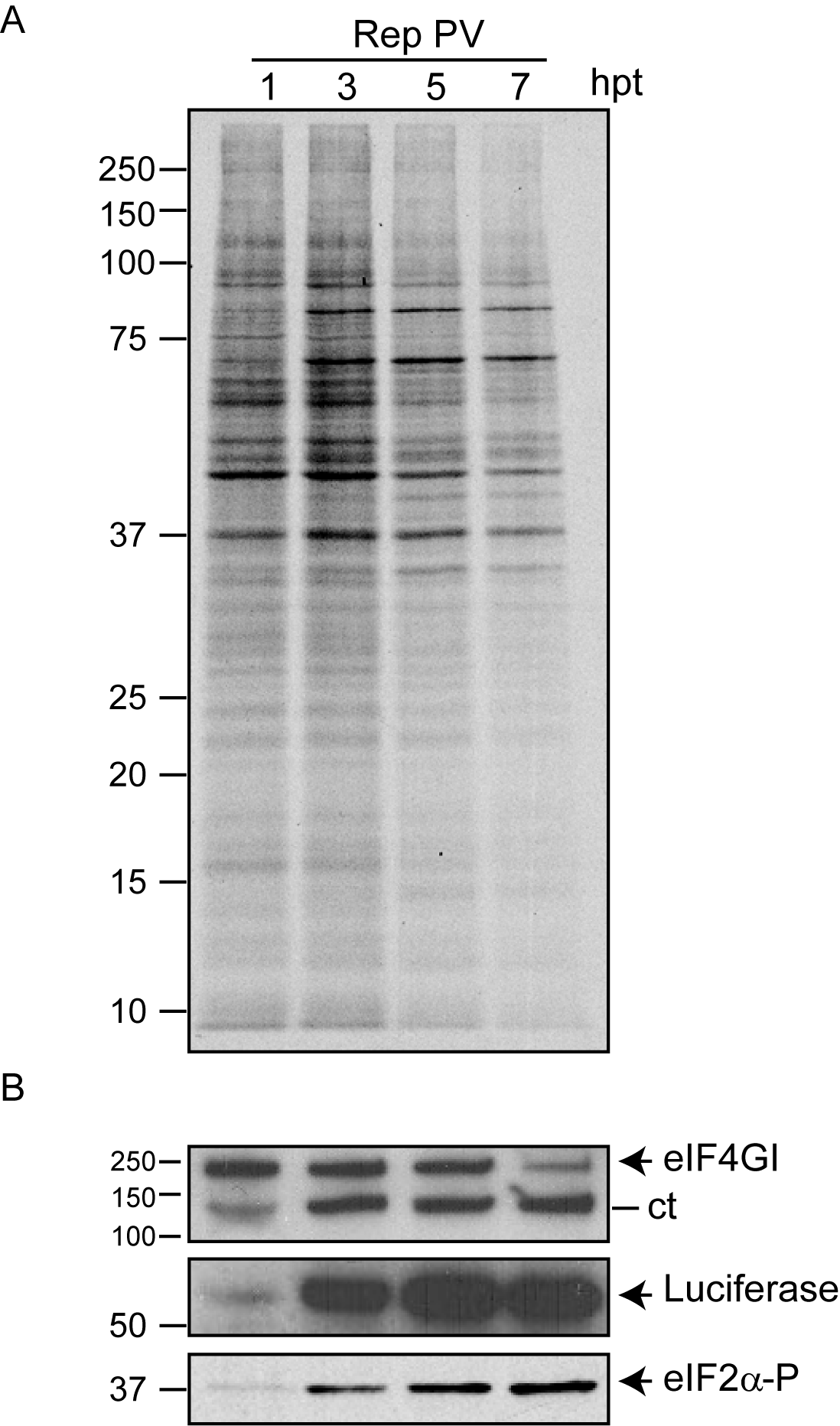

Supplement: Figure S1 — Kinetics of PV Replicon. BHKT7 cells were transfected withPV replicon. A) Protein synthesis was determined by labelling with [35S]Met-Cys for 45 minutes every two hours from 1 to 7 hpt. B) Western blot analysis of the samples obtained in panel A using anti-eIF4G, anti-Luciferase and anti-phospho-eIF2α. (TIF) [file pone.0025699.s001.tif]

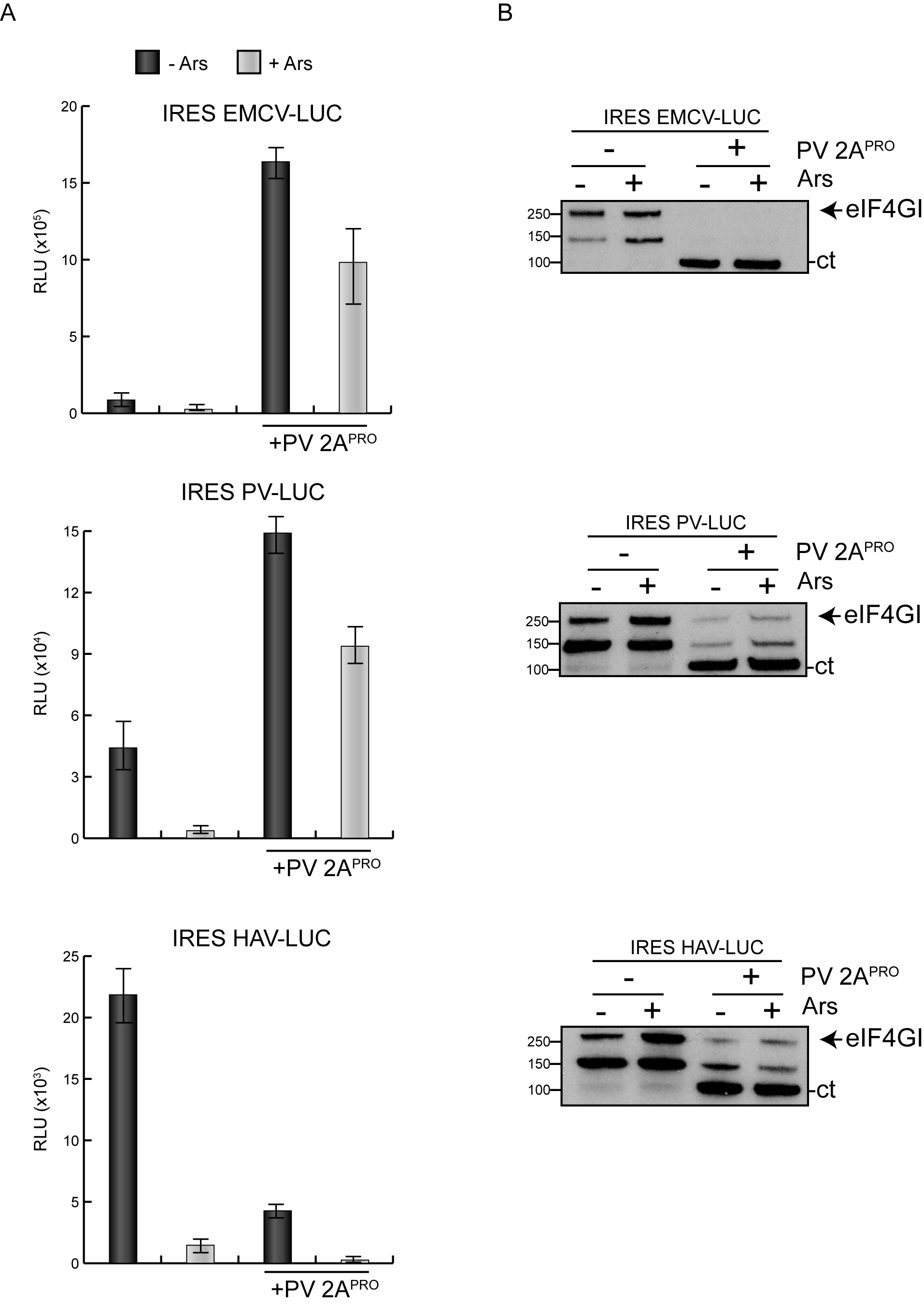

Supplement: Figure S2 — Rescue of picornavirus IRES translation by PV 2Apro in Huh7-T7 cells. A) Hepatoma cells were transfected with plasmids encoding EMCV IRES-luc, PV IRES-luc or HAV IRES-luc alone or co-transfected with pTM1-2A. At 2 hpt cells were treated or not with 200 µM Ars for 1 hour. Then, cells were harvested and lysated in luciferase buffer and luc activity was measured and represented as percentage from at least three independent experiments. Error bars indicate SD. B) eIF4GI were detected by western blot. (TIF) [file pone.0025699.s002.tif]

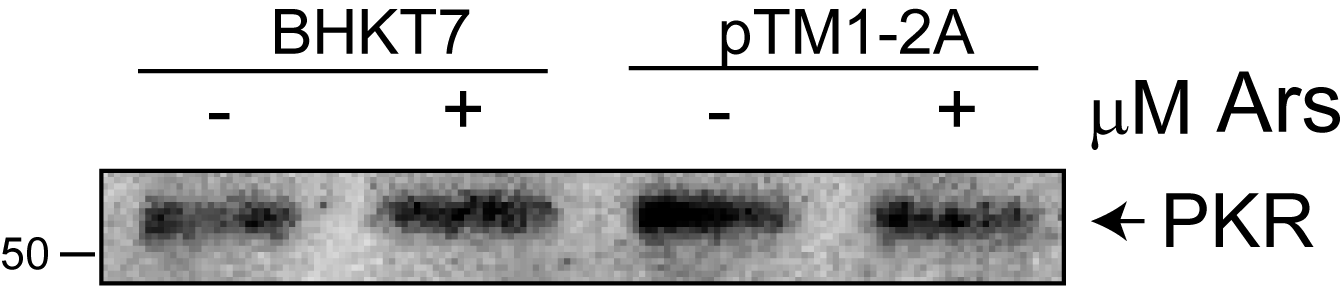

Supplement: Figure S3 — Effect of 2Apro on PKR. BHKT7 cells were mock- or transfected with pTM1-2A in presence or absence of Ars. Protein kinase RNA-activated (PKR) was detected by western blot. (TIF) [file pone.0025699.s003.tif]
